# Positive and negative reinforcement activate human auditory cortex

**DOI:** 10.3389/fnhum.2013.00842

**Published:** 2013-12-05

**Authors:** Tina Weis, Sebastian Puschmann, André Brechmann, Christiane M. Thiel

**Affiliations:** ^1^Biological Psychology Lab, Department of Psychology, European Medical School, Carl von Ossietzky UniversityOldenburg, Germany; ^2^Cluster of Excellence, “Hearing4all,” Carl von Ossietzky UniversityOldenburg, Germany; ^3^Special-Lab Non-invasive Brain Imaging, Leibniz Institute for NeurobiologyMagdeburg, Germany; ^4^Research Center Neurosensory Science, Carl von Ossietzky UniversityOldenburg, Germany

**Keywords:** feedback delivery, auditory cortex, reward, punishment, duration discrimination

## Abstract

Prior studies suggest that reward modulates neural activity in sensory cortices, but less is known about punishment. We used functional magnetic resonance imaging and an auditory discrimination task, where participants had to judge the duration of frequency modulated tones. In one session correct performance resulted in financial gains at the end of the trial, in a second session incorrect performance resulted in financial loss. Incorrect performance in the rewarded as well as correct performance in the punishment condition resulted in a neutral outcome. The size of gains and losses was either low or high (10 or 50 Euro cent) depending on the direction of frequency modulation. We analyzed neural activity at the end of the trial, during reinforcement, and found increased neural activity in auditory cortex when gaining a financial reward as compared to gaining no reward and when avoiding financial loss as compared to receiving a financial loss. This was independent on the size of gains and losses. A similar pattern of neural activity for both gaining a reward and avoiding a loss was also seen in right middle temporal gyrus, bilateral insula and pre-supplemental motor area, here however neural activity was lower after correct responses compared to incorrect responses. To summarize, this study shows that the activation of sensory cortices, as previously shown for gaining a reward is also seen during avoiding a loss.

## 1. Introduction

The ability to extract meaningful information from positive or negative outcomes of prior actions or preceding stimuli is a key requirement for learning. Prior studies in humans and animals compellingly demonstrate that sensory cortices develop increased responses to stimuli that gain behavioral relevance due to prediction of reward or punishment (e.g., Bakin et al., 1996; Thiel et al., 2002; Beitel et al., 2003; Thiel, 2003; Puschmann et al., 2013). Sensory plasticity is however only observed if a cognitive association is formed between the reinforcer and the sensory stimulus (Blake et al., 2006; Puschmann et al., 2013). Recently, several studies in humans have shown that even rewarding outcomes which follow the sensory stimuli activate respective sensory cortices in the absence of the respective sensory input (Pleger et al., 2008, 2009; Weil et al., 2010; Fitzgerald et al., 2013; Weis et al., 2013). In the studies by Pleger et al. (2008, 2009) participants had to discriminate somatosensory stimuli applied to an index finger and received a visually presented monetary reward for correct performance. Their results revealed increased neural activity in the somatosensory cortex contralateral to the judged hand after reward delivery. Using visual stimuli within a two-alternative forced-choice orientation-discrimination task in which correct discrimination resulted in an auditory reward, Weil et al. (2010) showed a similar effect within the visual cortex during feedback presentation. Similar results are seen in auditory cortex: Brosch et al. (2011) performed an auditory categorization task in monkeys and found that neural activity in auditory cortex reflected the reward expectancy and the received reward size. Weis et al. (2013) employed an auditory instrumental learning task in humans and similarly revealed evidence for increases in neural activity in auditory cortex during visual reward delivery in those trials where an expected reward was received and those trials where the expectation of obtaining no reward was correct. The enhancement of neural activity within auditory cortex was only seen in those participants who learned the paradigm. All together, those studies provide compelling evidence for sensory reactivation during positive reinforcement, but less is known with respect to negative reinforcement.

Different studies already investigated the effects of reward and punishment on learning and sensory representations. For example, Ilango et al. (2010) combined appetitive and aversive reinforcers in an auditory learning paradigm in Mongolian gerbils. Their data showed that punishment was more effective during initial learning, whereas reward was necessary to maintain a high level of conditioned responses. Kim et al. (2006) showed, in an instrumental choice task in humans, that avoiding an aversive outcome can even serve as a rewarding stimulus and that avoidance of aversive outcome recruits the same neural circuitries that are involved in reward processing. The effects of reward and punishment on neural activity to auditory stimuli were studied in ferrets, by David et al. (2012). The authors used an auditory instrumental learning task with a go/no go structure to test whether different behavioral responses (approach or avoidance) differentially impact neuronal responses to the same auditory target stimulus. Responses in auditory cortex were suppressed to the target sound in the approach condition and enhanced in the avoidance condition. Whether a similar differentiation would be seen in auditory cortex for the rewarding outcome which follows the sensory stimulation is unknown. We here aimed to investigate human auditory cortex activity at the time point of reinforcement under two conditions, positive and negative reinforcement. We used an auditory discrimination task, where participants had to judge the duration of frequency modulated tones. Correct performance was reinforced at the end of the trial, in one session by means of a financial gain and in another session by avoidance of financial loss. Given prior evidence that the activation of sensory cortices during reward outcome depends on the level of reward (Pleger et al., 2008, 2009; Weil et al., 2010; Brosch et al., 2011) the size of gains and losses was manipulated implicitly and could be either high or low depending on stimulus characteristics. Analysis of fMRI data focused on the time point of reinforcement delivery.

## 2. Materials and methods

### 2.1. Subjects

Twenty-six healthy normal volunteers (11 males, 15 females, age range = 20–29 years, average age = 24 ± 2 years) participated in the experiment. All participants were right-handed as indexed by a handedness inventory (Oldfield, 1971), had no history of neurological or psychiatric disease and had normal hearing (hearing loss less than 15 dB HL between 100 and 8 kHz). The study was conducted in accordance with the Declaration of Helsinki (World Medical Association, 2008). The experiments were approved by the ethics committee of the University of Magdeburg and written informed consent was obtained from the participants. Six participants had to be excluded because of severe head movements during fMRI measurements (head movement >3 mm).

### 2.2. Task

**Figure 1:**
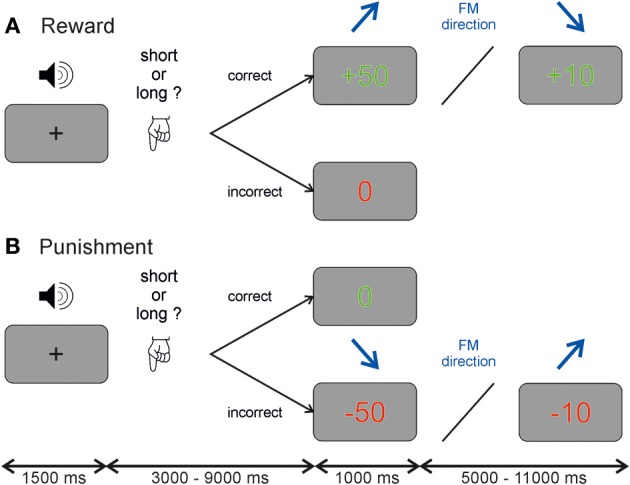
**Auditory discrimination paradigm**. Each participant performed a reward and a punishment session on two occasions. Each trial started with a frequency modulated tone and participants had to judge whether the tone was longer or shorter than 600 ms. Depending on the session participants received either **(A)** a reward of 10 or 50 Euro cent for correct answers and no money (indicated by a 0) for incorrect answers or **(B)** no money (indicated by a 0) for correct answers and a subtraction of either 10 or 50 Euro cent from a starting value of 25 Euros for incorrect answers. The value of the reinforcement depended in both cases on the direction of the frequency modulated tone which was unknown to the participant and randomized across subjects.

We used an auditory discrimination task, where participants had to judge whether an auditory stimulus (stimulus characteristics see below) was shorter or longer than 600 ms. The task was performed in a within-subject design under two reinforcement conditions, reward and punishment. The sessions were counterbalanced across participants and separated by 1–2 months to avoid learning effects. At the beginning of each trial participants heard a frequency modulated tone and had to categorize this tone by trial and error into either shorter (left button press using index finger of the right hand) or longer (right button press using middle finger of the right hand) than 600 ms. The duration of 600 ms was never presented to the subject. At the end of each trial a visual feedback was given to the participants. In the reward session correct answers were rewarded by either 10 or 50 Euro cent, which was presented on the screen as either “+10” or “+50” in green color. Incorrect answers were not rewarded which was indicated by a “0” in red color (see Figure 1A). During the punishment session incorrect answers were punished by subtracting 10 or 50 Euro cent from a fixed starting sum of 25 Euro, and shown on the screen as either “−10” or “−50” colored in red, whereas correct answers resulted in no loss indicated by a “0” marked in green color (see Figure 1B). Furthermore in this discrimination task there was an implicit conditioning included. The size of reward and punishment was linked to stimulus characteristics. Half of the participants received a high reward or punishment when the frequency modulated tone was ascending, the other half of participants for descending frequency modulated tones. This association was unknown to the participants (i.e., implicit conditioning).

**Figure A1:**
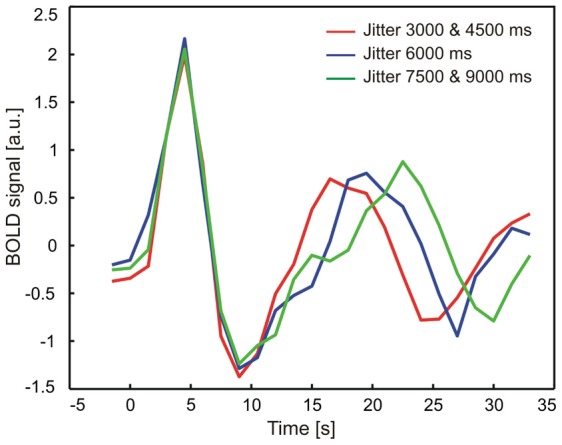
**Mean time course of neural activity in auditory cortex as a function of jitter between sound and reward presentation**. In a model-free approach, individual time courses were extracted and time locked to the sound presentation in a sphere of radius 6 mm around the peak maximum activation in the right auditory cortex [*x, y, z*] = [50, −2, 4]. Time courses were grouped according to different jitter lengths (red: 3000, 4500 ms, blue: 6000 ms, green: 7500, 9000 ms) to visualize the time course during sound as well as feedback presentation. The results of this analysis clearly showed that there is no major contamination of the measured brain activity during feedback presentation which resulted from the sound presentation.

A temporal jitter was used between the auditory stimulus and the reinforcement given at the end of the trial in steps of 1.5 s ranging from 3.0 to 9.0 s. The inter-trial-interval ranged from 5.0 to 11.0 s also in steps of 1.5 s. This temporal jitter allowed us to separate neural activity during auditory anticipation and reinforcement (see Figure A1). A fixation cross was presented in the middle of the screen when no visual stimulus was present. In each session, the experiment comprised 160 trials in 47 min. All experimental control software was programmed in MATLAB (The MathWorks, Inc., Natick, MA, USA) using Cogent 2000 (http://www.vislab.ucl.ac.uk/cogent.php). Participants received payment of the amount of gained reward or the remaining amount of money in the punishment session at the end of the experiment.

At the end of each scanning participants were presented with four different sounds from the experiment (short ascending, short descending, long ascending and long descending) and had to rate theses sounds according to pleasantness (1-pleasant till 5-unpleasant). Awareness of the contingencies was evaluated with a semi-structured interview. First, subjects were asked if they heard more ascending or descending FM tones during the experiment. Second they were asked whether they noted any relationship between the tones and the reinforcement-value and third, they had to select if either ascending or descending FM tones resulted in a higher reinforcement.

### 2.3. Stimuli

The auditory stimuli were frequency modulated tones with different stimulus dimensions (duration, direction, modulation rate, and frequency range). Sound duration was between 400 and 800 ms with steps of 10 ms, whereby a length of 600 ms served as reference, which was never presented to the participants. The modulation direction was either ascending or descending. Note that this was the stimulus dimension linked to the value of reinforcement. Modulation rate was either one or two octaves/second and there was a low and a high frequency band, each containing five onset frequencies separated by half-tone steps (500, 530, 561, 595, 630 Hz/1630, 1732, 1826, 1915, 2000 Hz). The sound levels were adjusted individually for each subject during a test scan until they reported that they could comfortably hear all stimuli.

### 2.4. fMRI data acquisition

FMRI data acquisition was performed on a 3 T Siemens MAGNETOM Verio MRI scanner (Siemens AG, Erlangen, Germany) with a twelve-channel head array. Key-presses were recorded using a MR-compatible response keypad (LUMITouch, Photon Control Inc., Burnaby, BC, Canada). Acoustic stimuli were delivered by MR compatible headphones (MR confon OPTIME 1, MR confon GmbH, Magdeburg, Germany).

During functional measurements 1885 T^*^_2_-weighted gradient echo planar imaging (EPI) volumes (time of repetition (*TR*) = 1.5 s, time of echo (*TE*) = 30 ms, flip angle α = 80°, field of view (FoV) = 200 × 200 mm^2^, voxel-size = 3.0 × 3.0 × 3.0 mm^3^) were obtained within one session. Note that subjects had to participate in two different sessions, reward and punishment, separated by 1–2 months. Volumes consisted of 27 slices (gap of 0.3 mm) ranging from the anterior cingulate cortex dorsally to the inferior colliculus in the brain stem. After the experimental task a high-resolution structural volume was obtained from each subject using a T_1_-weighted magnetization prepared rapid acquisition gradient echo (MPRAGE) sequence (*TR* = 1900 ms, *TE* = 2.52 ms, FoV 256 × 256 mm^2^, flip angle α = 9°, slice thickness = 1 mm, sagittal).

### 2.5. Behavioral data analysis

Discrimination accuracy as well as reaction times was analyzed for each participant and entered into repeated measurements ANOVAs with the factors *session* (reward/punishment) and *reinforcement-value* (high/low).

### 2.6. fMRI data analysis

MRI data were processed and analyzed using SPM8 (FIL, Wellcome Trust Centre for Neuroimaging, UCL, London, UK). To correct head motion, the functional time series were spatially realigned to the first image of the session. The structural T_1_-weighted volume was registered to the mean functional image and segmented in order to obtain spatial normalization parameters. Using these parameters, functional and structural images were normalized to the Montreal Neurological Institute (MNI) template brain. Finally, normalized functional volumes were smoothed with a three-dimensional Gaussian kernel of 4 mm full-width-half-maximum.

Single subject models were built separately for the reward and punishment session. Each single subject model contained four regressors of interest: two regressors for BOLD responses to correct and incorrect trials, for both time points within the experiment, the anticipation (sound presentation) and reinforcement (feedback presentation) phase. For each of the regressors we added a parametric modulation for the different reinforcement-values by including either +1 for a high reward/low punishment or −1 for low reward/high punishment. Further, signal changes related to head movement were accounted for by including the six movement parameters as calculated in the SPM8 realignment procedure as additional regressors. Time series in each voxel were high-pass filtered to 1/128 Hz and modeled for temporal autocorrelation across scans with an AR(1) process.

Statistical data analysis was focused on neural responses to frequency modulated tones during reinforcement. Single subject contrasts coding for correct and incorrect trials during feedback presentation were entered into a flexible factorial ANOVA design with the following factors: *subject, session* (reward/punishment), and *correctness* (correct/incorrect). Within this ANOVA we calculated both main effects (*session* and *correctness*) as well as the *session x correctness* interaction. Furthermore, we calculated a paired *t*-test between the reward and punishment session with respect to the effects of parametric modulation, pooling over correct and incorrect trials. Results of these analyses were thresholded at a single voxel value of *p* < 0.001 and are reported corrected for the whole brain or for the right and left auditory cortex as region of interest at *p* < 0.05, established with a Monte Carlo voxel-cluster threshold technique (see program AlphaSim by Douglas Ward in AFNI software [http://afni.nimh.nih.gov/pub/dist/doc/manual/AlphaSim.pdf; Cox (1996)]. All clusters were identified using a corrected alpha level of 0.05 (voxelwise *p* < 0.001; cluster-size ≥110 voxels, for total scanning volume; cluster-size ≥18 voxels for small volume correction, indicated by asterisks). To further visualize significant effects, we extracted averaged beta values as a function of correctness and session in a sphere of radius of 6 mm around the activation peak maxima in different regions. Note that this type of data visualization does not contain circularity effects according to Kriegeskorte et al. (2009); Vul et al. (2009) since we used a flexible-factorial ANOVA and afterwards determined the source of significance within a main effect or interaction. This approach is an extension of the same analysis and not double dipping. Note that the extraction of beta values was only illustrative and inferences were taken from the original analysis.

#### 2.6.1. Functional localizer

In the second fMRI session a functional localizer was acquired after the end of the task. This localizer aimed at identifying brain regions responsive to frequency modulated tones. Subjects were presented with 28 blocks of frequency modulated tones (20 s), which were interleaved by 10 s of silence and had to judge either the *duration* (short/long) or the *direction* (ascending/descending) of the tones by pressing the left button for short or decreasing tones and the right button for long or increasing tones, respectively. Frequency modulated tones were presented every 2 s and varied in the same stimulus dimensions described above, apart from duration, which was either 400 or 800 ms. Each condition (judging the duration or direction) lasted for seven consecutive blocks before it switched to the other condition. There was no feedback given to the participants and they only saw a fixation cross during the whole measurement with either a short and long or upward and downward arrow, to indicate the task. 705 scans were acquired with the same scanning parameters as above.

For each subject we modeled the short and long as well as ascending and descending tones separately and also included the movement parameters, which resulted in a single subject model with 10 regressors. At group level we used the contrast *tone > silence* masked with the superior temporal gyrus and Heschl's gyrus (as included in the WFU PickAtlas extension for SPM (Maldjian et al., 2003), *p* < 0.001 uncorrected) as region of interest for the correction of the results within the main paradigm.

## 3. Results

### 3.1. Behavioral data

Discrimination accuracy was similar in the reward and punishment session (% correct responses reward: 77.59 ± 0.26, % correct responses punishment: 78.31 ± 0.17, *T*_(1, 19)_ = −1.3031, *p* = 0.20). The mean reward over all subjects was 37.49 ± 2.37 Euro, whereas the mean remaining money within the punishment session was 14.35 ± 2.02 Euro. Analysis of variance revealed no significant effect neither between *session* or *reinforcement-value*, nor a *session x reinforcement-value* interaction.

In contrast reaction times showed a significant interaction between *session* and *reinforcement-value* [*F*_(1, 19)_ = 5.72, *p* = 0.027]. Participants reacted slower in those trials with a high punishment (1355.8 ± 310.72 ms) compared to the low punishment (1288.8 ± 219.57 ms) and vice versa for high and low reward in the reward session (high reward: 1272.5 ± 211 ms, low reward 1289.7 ± 204.16 ms). Note that there was no main effect of *session* nor *reinforcement-value*.

Within the semi-structured interview, none of the participants noticed any relationship between reinforcement-value and the features of the FM tones. The rating whether the ascending or descending FM tones resulted in a higher reward was around chance level (40%).

To test for implicit conditioning, we analyzed pleasantness ratings to ascending and descending frequency modulated tones which were differentially associated with high and low reward and punishment. The results of a *t*-test revealed no difference between the ratings for tones with high and low reward [*T*_(1, 39)_ = −0.54, *p* = 0.58] or high and low punishment [*T*_(1, 39)_ = 0.18, *p* = 0.85].

### 3.2. fMRI data—main effect of correctness

**Figure 2:**
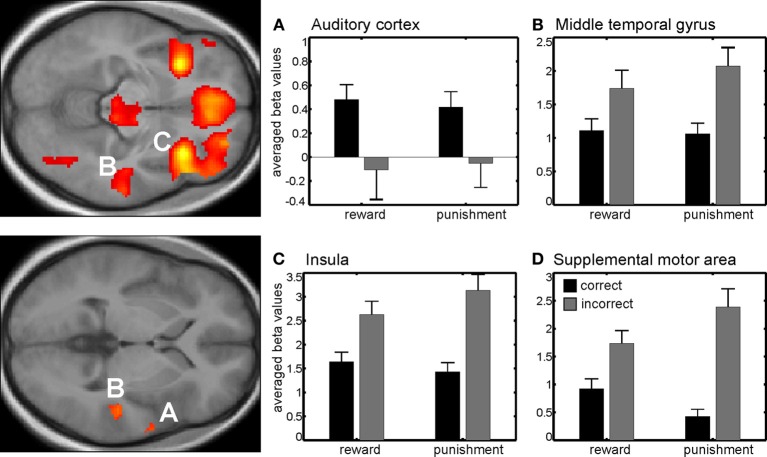
**Main effect of correctness**. Beta values in right auditory cortex **(A)** show a higher neural activity for correct trials compared to incorrect trials. Other brain areas showing differential responses were the right middle temporal gyrus **(B)**, the right insula **(C)**, and the pre-supplemental motor area **(D)** among others. Note that here differences are due to higher neural activity in incorrect compared to correct trials. Activations are superimposed on the mean of the individual subject T_1_ images for at *p* < 0.001 (uncorr., *k* > 110 voxels). Note that the extraction of beta values is only illustrative and inferences were made from the original analysis.

During reinforcement, we found a main effect of correctness in right auditory cortex which was due to higher activity for correct compared to incorrect trials for both reinforcement types. In other words, the auditory cortex was responsive to either obtaining a reward or avoiding a punishment after a correct discrimination was made. Other regions showing a main effect of correctness were the right insula, the supplemental motor area and the right middle temporal lobe. Note however that here the effect was due to an enhanced response to incorrect compared to the correct trials, i.e., when no reward was obtained or when a punishment occurred after making a mistake (Figure 2, Table A1A).

### 3.3. fMRI data—interaction session x correctness

**Figure 3:**
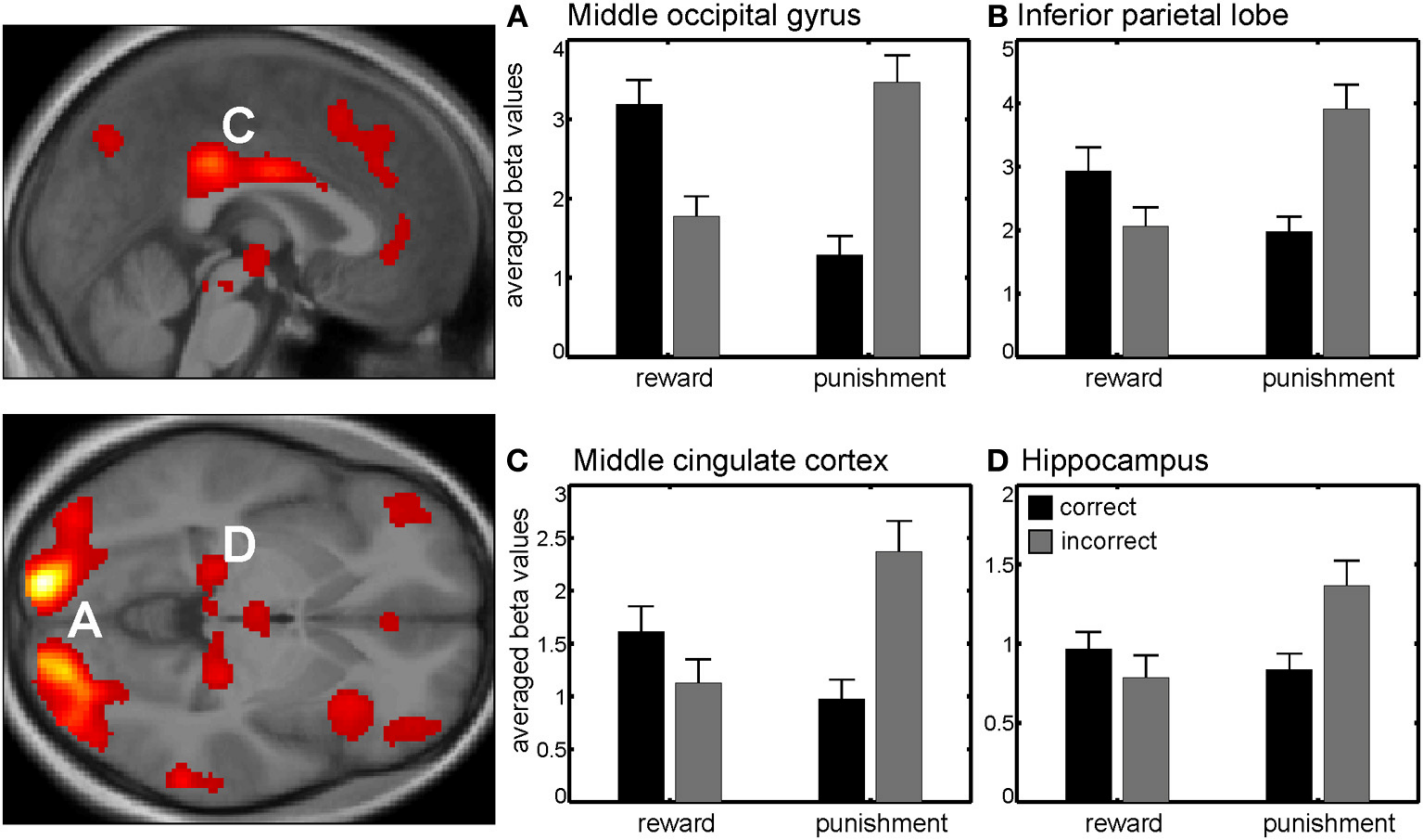
**Interaction between session and correctness**. Beta values in right middle occipital gyrus **(A)** left inferior parietal lobe **(B)** middle cingulate cortex **(C)** and right hippocampus **(D)** revealed higher activity in those trials with higher valence (either negative or positive), i.e., when gaining money in the reward session or losing money in the punishment session. Activations are superimposed on the mean of the individual subject T_1_ images for at *p* < 0.001 (uncorr., *k* > 110 voxels). Note that the extraction of beta values is only illustrative and inferences were made from the original analysis.

Additionally, we found a significant interaction between session and correctness within the bilateral middle occipital gyrus, bilateral inferior parietal lobe, middle cingulate cortex and bilateral inferior frontal gyrus (Figure 3, Table A1B). Beta values indicated that this interaction reflected increased neural activity when either a reward or punishment occurred, i.e., after correct discrimination in the reward session and incorrect performance in the punishment session.

### 3.4. fMRI data— effects of reinforcement-value

**Figure 4:**
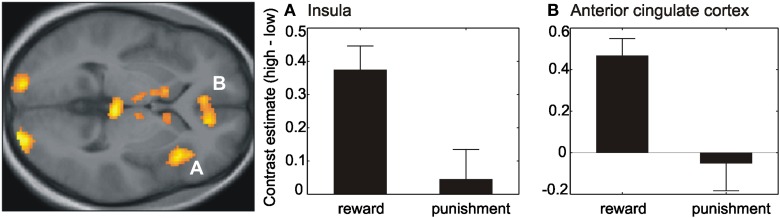
**Effect of reinforcement-value**. Contrast estimates (high–low) within the right anterior insula **(A)** and anterior cingulate cortex **(B)** revealed a difference between the high and low reinforcement within the reward session but no difference within the punishment session. Activations are superimposed on the mean of the individual subject T_1_ images at *p* < 0.001 (uncorr., *k* > 110 voxels). Note that the extraction of contrast estimates is only illustrative and inferences were made from the original analysis.

Results of the paired *t*-test between reward and punishment session with respect to the effects of different reinforcement-values resulted in a higher activation during reward in contrast to punishment within the right and left visual cortex as well as the anterior cingulate cortex and right insula (Table A2, Figure 4). These regions showed a higher activity for high versus low reinforcement.

## 4. Discussion

Our findings provide new evidence that, during reinforcement, human auditory cortex is similarly activated by reward and avoidance of punishment. This activation was not modulated by reinforcement value. A modulation by reinforcement value was mainly found in the reward session and occurred in anterior cingulate cortex and right anterior insula among others.

### 4.1. Neural activity following reinforcement

During reinforcement we found a higher activity for correct compared to incorrect trials within the right auditory cortex. The peak maximum activation is at the same location as described in Weis et al. (2013) using positive reinforcement only and an operant conditioning task. Here, we confirm that reinforcement-induced activation of auditory cortex in absence of any auditory stimulus is also seen in an auditory discrimination task. These results are in line with findings in somatosensory (Pleger et al., 2008, 2009) and visual discrimination tasks (Weil et al., 2010). The important new result is however the finding that sensory cortices are similarly responsive to gaining a reward and avoiding a loss, since at least in auditory cortex we found an increase in neural activity when a reward was obtained in the reward session or a punishment was avoided in the punishment session. None of the previously mentioned studies investigated the effects of punishment on reactivation in the sensory cortices. Note that the opposite neuronal responses in auditory cortex under approach and avoidance conditions in the study of David et al. (2012) where recorded at the time point of auditory target presentation. Further, motor contingencies in our study were similar for the approach and avoidance condition and did not involve behavioral inhibition.

We also found several brain regions with higher activity for incorrect compared to correct trials, e.g., right middle temporal gyrus (BA 21), pre-supplemental motor area (SMA), and bilateral anterior insula. The middle temporal cortex (BA 21), with higher activation for incorrect compared to correct trials has been shown to be activated during voluntary attention shifts to infrequent sounds (Sabri et al., 2006; Huang et al., 2012). A duration discrimination study by Sabri et al. (2006) in humans, suggests that the middle temporal cortex exhibits higher activity to difficult compared to easy trials. This is in line with our finding of higher activity in incorrect compared to correct trials, since most mistakes were made when tone duration was close to 600 ms. The same activation pattern is also seen within the pre-SMA as well as the bilateral insula. Both regions have previously been linked to error processing, especially pre-SMA as a source region for error-related negativity in EEG studies (Scheffers et al., 1996; Holroyd et al., 2004, 2006; Taylor et al., 2007). There are also fMRI studies investigating feedback related activity within pre-SMA with higher responses to negative compared to positive feedback (Ullsperger and von Cramon, 2004; Özyurt et al., 2012) as well as to omitted or delayed compared to immediate feedback (Kohrs et al., 2012).

We also found brain regions showing an interaction between session and correctness, as for example the bilateral middle occipital gyrus (BA 17) and the middle cingulate cortex (BA 23). All regions revealed a higher activity for those trials with higher valence (either negative or positive), in other words, the gain trials within the reward session and the loss trials within the punishment session. Another region activated in this contrast was the hippocampus. Shigemune et al. (2013) provide evidence that memory is enhanced by the motivation of avoiding punishments and could be modulated by interactions between brain regions associated with the prediction of punishments such as the ventral tegmental area/substantia nigra, nucleus accumbens, or insula and the hippocampus, which is involved in memory (Adcock et al., 2006; Murty et al., 2012).

### 4.2. Effects of reinforcement-value

With respect to the value of reinforcement, we found no differences within the auditory cortex. A previous study by Pleger et al. (2008), using a comparable discrimination paradigm involving the somatosensory cortex, showed an effect of different reward sizes on the reactivation of the sensory cortex during feedback presentation. However, in contrast to the implicit conditioning in our study, participants in the study by Pleger and colleagues (2008) were aware of the reinforcement values since this was presented at the beginning of each trial.

Several other brain regions, such as anterior cingulate cortex and right anterior insula were however responsive to the value of reinforcement, even though this was implicitly manipulated and participants were not aware of the contingencies. Extracting the mean beta values revealed that the difference here was mainly driven by the reward session with a higher activity for high rewarded (+50) compared to low rewarded (+10) trials, whereas there was almost no difference within the punishment session. Note however, that the number of trials where a punishment was obtained after incorrect performance was much lower than the number of trials where a reward was obtained after correct performance.

### 4.3. fMRI data on reward and punishment

Several regions revealed a main effect of correctness, i.e., similar brain activity to obtaining a financial reward or avoiding a financial punishment. In contrast to most other studies involving appetitive and aversive reinforcement, we measured both sessions separately. Within the reward session participants had the possibility to gain a reward for correct performance at the end of each trial, but were not punished for incorrect answers. In the punishment session, participants lost money for incorrect answers but on the other hand, could not gain any reward for correct answers. Probably due to this separation, the positive outcome in the punishment session leads to the same reaction as the positive outcome in the reward condition. This was already suggested by Kim et al. (2006) who found that avoiding an aversive outcome leads to the same activation as reward itself. Also Palminteri et al. (2012) revealed some evidence, that testing punishment in a separate session as reward can shift the neural activity such that not being punished serves as rewarding and hence recruits reward instead of punishment areas. Using a simple monetary gambling task, Nieuwenhuis et al. (2005) showed that reward processing systems determine an outcome as favorable or unfavorable on the range of possible outcomes, regardless of the absolute magnitude of the outcomes. However, even if there is no difference between obtaining reward and avoiding a punishment within this study, our results always show a numerically larger difference between correct and incorrect trials within the punishment session. Hence punishment might result in slightly larger differential activity. In Mongolian gerbils using a combination of appetitive and aversive reinforcers, Ilango et al. (2010) found that the effect of appetitive reinforcers typically saturates with prolonged presentation while the effect of aversive reinforcers does not. Furthermore, the motivation of avoiding punishments might be slightly higher than the motivation of receiving rewards (Seymour et al., 2007).

## 5. Conclusion

In summary our findings in auditory cortex underline its role in higher cognitive processes. We here show in an auditory discrimination task with positive and negative reinforcement that the auditory cortex is not only responsive to rewards but also to avoiding punishment at the time point of feedback presentation.

### Conflict of interest statement

The authors declare that the research was conducted in the absence of any commercial or financial relationships that could be construed as a potential conflict of interest.

## Appendix

**Table A1:** **Brain regions showing neural activity during reinforcement in main effect of correctness (A) and interaction session x correctness (B)**.

	***x***	***y***	***z***	**Volume**	***Z***	**Region**
(A)	50	−2	4	23	3.38	Auditory cortex R^*^
	64	−20	8	27	3.45	Auditory cortex R^*^
	50	−24	−6	713	5.67	Middle temporal lobe R
	−32	20	−8	2560	7.37	Insula L
	48	22	8	5389	7.28	Insula R
	4	22	54	3647	7.05	Pre−Supplemental motor area
	2	−24	28	466	6.07	Middle cingulate cortex R
	40	−22	56	3551	6.05	Precentral R
	50	−50	52	1078	5.80	Supramarginal R
	−62	−48	38	282	5.48	Supra marginal L
	−6	44	−12	1614	5.78	Frontal middle orbital L
	−20	24	46	985	5.56	Frontal middle L
	−44	−72	28	520	5.40	Angular L
	−6	−52	14	813	5.27	Precuneus L
	10	−66	38	275	5.33	Precuneus R
	8	−6	6	1979	4.99	Thalamus R
	38	−64	−14	132	3.98	Fusiform gyrus R
	−38	−50	44	149	3.90	Inferior pariental gyrus L
	34	−18	20	200	3.89	Rolandic operculum R
(B)	−16	−96	−2	3184	>8	Middle occipital gyrus L
	26	−84	−8	2955	7.46	Middle occipital gyrus R
	32	−64	44	5797	7.04	Inferior parietal lobe R/L
	2	−32	32	1289	6.37	Middle cingulate cortex R
	6	18	44	1340	4.33	Middle cingulate cortex R
	−42	4	26	1810	5.36	Inferior frontal gyrus L
	50	42	16	3634	6.58	Middle frontal gyrus R
	20	−30	−2	321	5.70	Hippocampus R
	−18	−32	−2	187	4.50	Hippocampus L
	−2	−14	−6	190	3.93	Thalamus L

All regions reported using a corrected alpha level of 0.05 (voxelwise p < 0.001; cluster-size ≥110 voxels, for total scanning volume; cluster-size ≥18 voxels for small volume correction, indicated by asterisks).

**Table A2:** **Brain regions showing differential neural activity for high and low reinforcement-values**.

***x***	***y***	***z***	**Volume**	***Z***	**Region**
4	36	18	2953	5.39	Anterior cingulate cortex
34	24	−4	390	4.46	Right anterior insula
24	−98	−2	266	4.47	Right visual cortex
−16	−96	−4	355	5.10	Left visual cortex

For all regions reported we used a corrected alpha level of 0.05 (voxelwise p < 0.001; cluster-size ≥110 voxels, for total scanning volume; cluster-size ≥18 voxels for small volume correction, indicated by asterisks).
